# Productivity and Efficiency of a Department Resident Aesthetic Plastic Surgery Clinic

**DOI:** 10.1093/asjof/ojac084

**Published:** 2022-12-06

**Authors:** Hani Y Nasr, Carter J Boyd, Zachary M Borab, Neil M Vranis, Michael F Cassidy, Alexis K Gursky, Rebecca Gober, Barry M Zide, Daniel J Ceradini

**Affiliations:** From NYU Langone Health, Hansjörg Wyss Department of Plastic Surgery, New York, NY, USA; From NYU Langone Health, Hansjörg Wyss Department of Plastic Surgery, New York, NY, USA; From NYU Langone Health, Hansjörg Wyss Department of Plastic Surgery, New York, NY, USA; From NYU Langone Health, Hansjörg Wyss Department of Plastic Surgery, New York, NY, USA; From NYU Langone Health, Hansjörg Wyss Department of Plastic Surgery, New York, NY, USA; From NYU Langone Health, Hansjörg Wyss Department of Plastic Surgery, New York, NY, USA; From NYU Langone Health, Hansjörg Wyss Department of Plastic Surgery, New York, NY, USA; From NYU Langone Health, Hansjörg Wyss Department of Plastic Surgery, New York, NY, USA; From NYU Langone Health, Hansjörg Wyss Department of Plastic Surgery, New York, NY, USA

## Abstract

**Background:**

There has been increasing demand for aesthetic surgery procedures in the United States, highlighting the critical importance of the competence of plastic surgery residents and rigorous methods of aesthetic surgery training.

**Objectives:**

The objective of this study was to review procedures and outcomes from our plastic surgery resident aesthetic clinic. Outcomes and costs were compared to national averages and reports from the literature.

**Methods:**

A retrospective chart review identified all adult patients who presented to the Resident Aesthetic Surgery Clinic at NYU Langone Health in 2021. Patient demographics, comorbidities, procedural data, postoperative complications, revisions, and surgeon fees were compiled. A brief confidence survey was distributed to participating residents before and after their clinic rotation. Data were analyzed using IBM SPSS software (Armonk, NY).

**Results:**

In 2021, 144/379 consultations led to an operation (38.0% conversion rate), resulting in 420 distinct surgical procedures. The majority (53.3%) of procedures involved the head and neck. Complication and revision rates were 5.5% and 1.0%, respectively, with surgeon fees consistently below the national average. Residents reported being significantly more confident performing face lifts, rhinoplasties, and aesthetic surgery in general following their clinic rotation.

**Conclusions:**

These data represent the largest annual reported study of plastic surgery resident aesthetic procedures and outcomes, demonstrating the high volume and productivity of the NYU Resident Aesthetic Surgery Clinic. These results further support resident aesthetic clinics as a robust training modality.

**Level of Evidence: 4:**

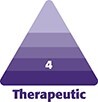

Over the past several decades, the number of aesthetic procedures in plastic surgery has risen drastically in the United States. Despite the COVID-19 pandemic, aesthetic procedures have increased 54% in the last year alone, surmounting $10 billion in annual revenue.^1^ To meet this public demand, it has been more important than ever to ensure plastic surgery residents are well equipped to incorporate aesthetics into their future practice. Their training, however, has not been without challenges. Given the elective nature of aesthetic surgery, many patients opt to personally select their surgeon from qualified private practitioners. Patients’ tendency to avoid academic institutions, coupled with relatively high postoperative expectations, has historically led to limited hands-on experience for residents. As a result, graduating plastic surgery residents have felt deficient in performing aesthetic procedures, particularly those of the face.^2,3^ Furthermore, in a recent survey, nearly 30% of department chairs/division chiefs acknowledged difficulties with and inadequacies of aesthetic surgery training at their institutions and recommended the need for more effective training models.^4^

Resident-directed aesthetic surgery clinics are in place to provide a more comprehensive experience to trainees. Programs use a discounted fee-for-service compared to private practice fee schedules, while still offering the expertise of a supervising attending surgeon in order to attract patients to these clinics. It was not until 2014, however, that the Accreditation Council for Graduate Medical Education (ACGME) increased the minimum required aesthetic cases from 50 to 150 for graduating plastic surgery residents.^5^ Only recently have we seen the effect of this increased caseload on resident preparedness and patient outcomes. Resident aesthetic clinics have evolved into a robust educational modality and account for a substantial portion of resident exposure to aesthetic cases, while providing safe and successful outcomes.^6^ Additionally, these clinics further enhance resident autonomy, decision-making, and surgical maturity.^2^

The Resident Aesthetic Surgery Clinic at the Hansjörg Wyss Department of Plastic Surgery at NYU Langone Health has been in existence for the past 2 decades. Recently, the clinic underwent significant restructuring with a focus based on amplifying an intensive resident experience specifically in areas that are deficient on a national training level and consistent with the patient aesthetic demand. With continual departmental support, the clinic maintains a high procedural volume while providing care for patients of varying complexities to closely recapitulate local aesthetic surgery practice patterns. Prior studies have evaluated institution-specific experiences with resident aesthetic clinics but were frequently limited by small patient cohorts. Thus, despite several reports, there remains a paucity of literature evaluating the overall surgical output of this training mechanism.

In the current study, we present an entire year of data from the NYU Aesthetic Clinic which represents the highest volume experience of supervised resident aesthetic clinics in the literature when accounting for the duration of the study. The purpose of this analysis is to demonstrate the surgical productivity and efficiency of this clinic in the 2021 calendar year and to highlight its impact on graduating plastic surgery residents. Overall, we demonstrate that the NYU Aesthetic Clinic is a cohesive model that provides superior care for patients through a collaborative effort from residents, attendings, clinic staff, and administrators.

## METHODS

### Aesthetic Surgery Clinic Structure

The Resident Aesthetic Surgery Clinic at the Hansjörg Wyss Department of Plastic Surgery at NYU Langone Health sees patients 5 days per week and provides a total of 6 months of training experience for residents: 3 months during the postgraduate year (PGY)-5 and 3 months during PGY-6. During this rotation, residents solely dedicate their time to the resident aesthetic clinic. If they are not occupied with cases, residents have the ability to supplement their training by scrubbing into attending cases (data not included in this study). Patients presenting to the clinic are first evaluated by residents, who perform the entire consultation. Patients are staffed with the attending present that day who provides feedback, oversight, and supervision and helps the residents refine their clinical skills of indicating a patient for surgery, preoperative planning, and postsurgical follow-up visits. While 2 dedicated attending faculty provide the majority of coverage, all throughout the department of plastic surgery are available to residents for additional supplementary education or staffing a case that may be within their expertise. Once a patient establishes a patient–doctor relationship with a specific resident, that same resident will follow the patient throughout the remainder of their residency training. If a procedure or follow-up appointment is scheduled after a resident has completed their dedicated aesthetics rotation, that resident has the flexibility among their other rotations to follow the patient longitudinally in the aesthetic clinic and continue to provide surgical and postsurgical care to their patient. As a result, in each resident accumulates their own patient population and provides longitudinal care beyond the duration of their aesthetic clinic rotation.

**Figure 1.**
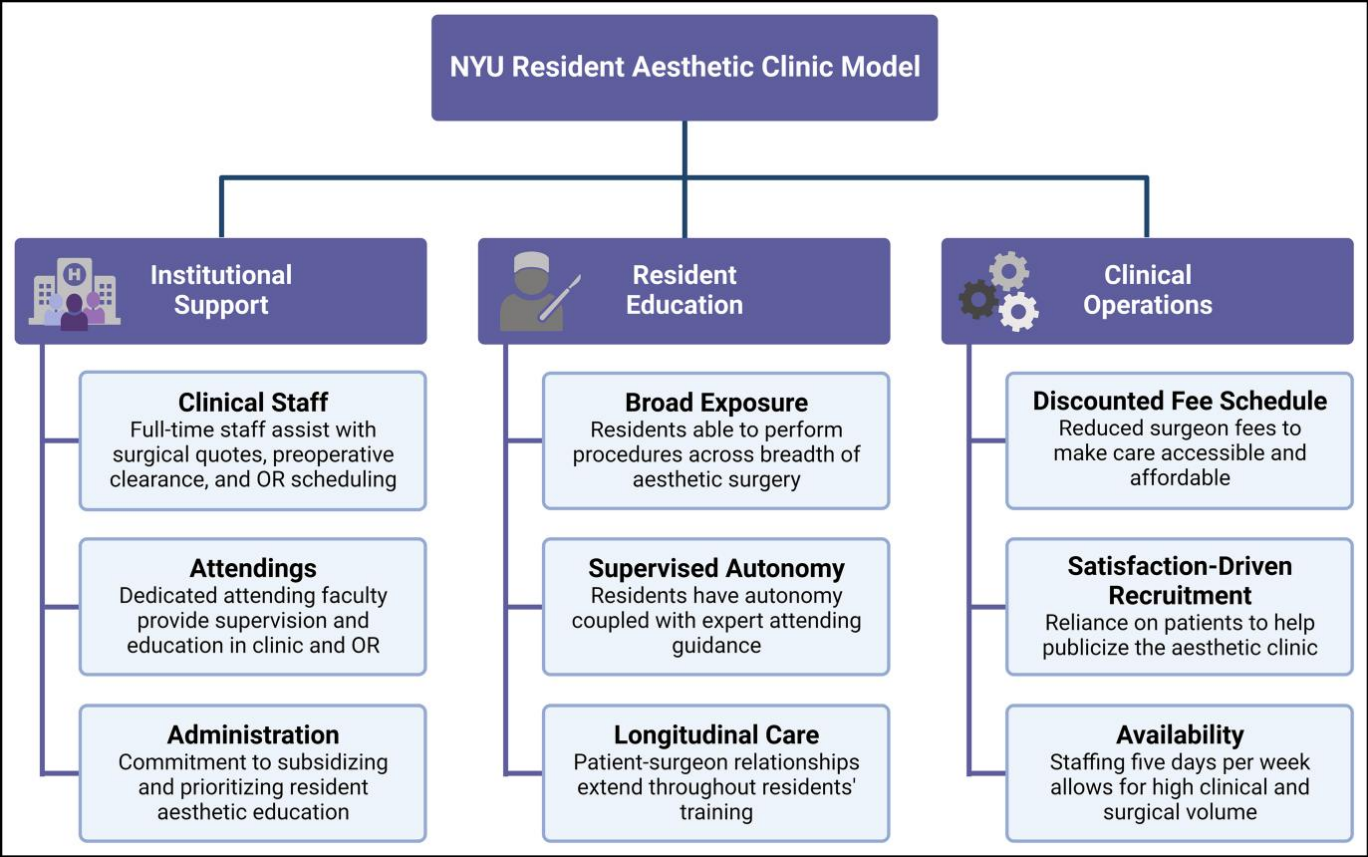
Diagram depicting the cohesive structure of the NYU Resident Aesthetic Clinic. Three fundamental pillars include institutional support, resident education, and clinical operations. Created with and published with permission from BioRender.com.

Dedicated clinic front office staff streamline the process by assisting with surgical quotes, preoperative clearance, and operating room scheduling. The close proximity of the operating room suite to the faculty plastic surgery practices (same or one floor above) allows for leveraging resources to ensure that the clinic is well staffed and supervised. All surgical follow-ups are seen by residents under attending supervision. The resident aesthetic clinic does not engage in any formal advertising, but rather relies on word-of-mouth and direct referrals for patient recruitment. Patients are billed on a fee-for-service basis and insurance is not accepted. The revenue generated is used to offset operational costs of the clinic and operating rooms, as well as contribute to the salary of staff supporting the clinic. The remaining operational costs of the clinic are subsidized by the department. Key pillars of our resident aesthetic clinic model are highlighted in Figure 1.

### Study Design

We conducted a retrospective review of all surgical procedures completed by the NYU Resident Aesthetic Surgery Clinic in 2021. This study adhered to protocols approved by the Institutional Review Board (IRB), in accordance with the Code of Federal Regulations on the Protection of Human Subjects (45 CFR Part 46). Written consent was provided, by which the patients agreed to the use and analysis of their data. Patient demographics and procedure information from the electronic medical record were tabulated. Patients were excluded from the study if surgical outcome information was missing. Participating residents were surveyed before and after their rotation in the clinic to assess their confidence in performing aesthetic procedures which was rated on a scale of 1 to 5 (Supplemental Table 1).

### Procedures and Complications

Procedures were defined similarly to how procedures are recorded for resident case logs for the Accreditation Council for Graduate Medical Education (ACGME).^5^ Bilateral procedures (eg, bilateral breast augmentation) were counted as 2 separate procedures (left and right). Blepharoplasty was not only separated by laterality but also by upper and lower lids. In contrast, an operation was defined as a single trip to the operating room or office visit in which the patient received at least 1 procedure. As an example, a patient who received bilateral upper and lower blepharoplasties concurrently was considered to have 4 separate procedures during 1 operation. Complications were categorized as either minor, moderate, or major. Minor complications were treated with nonoperative management. Moderate complications required a small intervention such as seroma aspiration, while major complications necessitated a return to the operating room. Complication and revision rates were calculated by summing the number of complications and revisions, respectively, and dividing by the total number of procedures.

### Data Analysis

Data were organized and analyzed using IBM SPSS software and the ggplot2 package in R.^7^ A chi-squared test assessed for significant deviation in frequencies of procedures across yearly quarters. Mann–Whitney tests evaluated the significance between pre- and postrotation confidence ratings among residents. A *P*-value of <0.05 was considered statistically significant. Complication and revision rates were calculated with respect to the total number of procedures. The complication rate, revision rate, and surgeon fees were compared to data from other institutions^8–12^ available in the literature. Surgeon fees were also compared to national averages according to the 2020 ASPS Plastic Surgery Statistics Report^13^ and 2021 Aesthetic Plastic Surgery National Databank Statistics.^1^

## RESULTS

### Aesthetic Surgery Clinic Patients Tend to Be Middle-aged Females in Good Overall Health

In the year 2021, 132 patients underwent an operation at the NYU resident aesthetic surgery clinic. These patients were predominantly female (male to female ratio ∼1:18) and in relatively good health in-line with national trends,^1^ with the most common comorbidity being hypertension found in 9.8% of patients (Table 1). With respect to risk factors for wound healing, diabetes and active tobacco use were seen in only 0.8% and 3.0% of patients, respectively. Additionally, the average age of patients on their date of surgery was 52.3 ± 13.0 years old (range 23.2-77.3) and the average BMI was 25.5 ± 4.2 kg/m^2^.

**Table 1.** Patient Demographics and Comorbidities

Patient demographics	No. (%)
Gender	
Female	125 (94.7)
Male	7 (5.3)
Age	
<30	12 (9.1)
30-49	51 (38.6)
50-69	57 (43.2)
≥70	12 (9.1)
BMI^a^	
Underweight (<18.5)	4 (3)
Healthy weight (18.5-24.9)	63 (47.7)
Overweight (25-29.9)	35 (26.5)
Obese (30-39.9)	24 (18.2)
Comorbidities	
Hypertension	13 (9.8)
Hypothyroidism	8 (6.1)
Anxiety	6 (4.5)
Asthma	5 (3.8)
Tobacco use, current	4 (3)
Cancer, history	4 (3)
Hyperlipidemia	4 (3)
Coronary artery disease	2 (1.5)
Chronic kidney disease	2 (1.5)
Depression	1 (0.8)
Diabetes	1 (0.8)

Patients’ gender, age, BMI, and comorbidities shown. ^a^Data unavailable for 6 patients.

### Head and Neck Procedures Are the Most Frequently Performed in the Aesthetic Surgery Clinic

**Figure 2.**
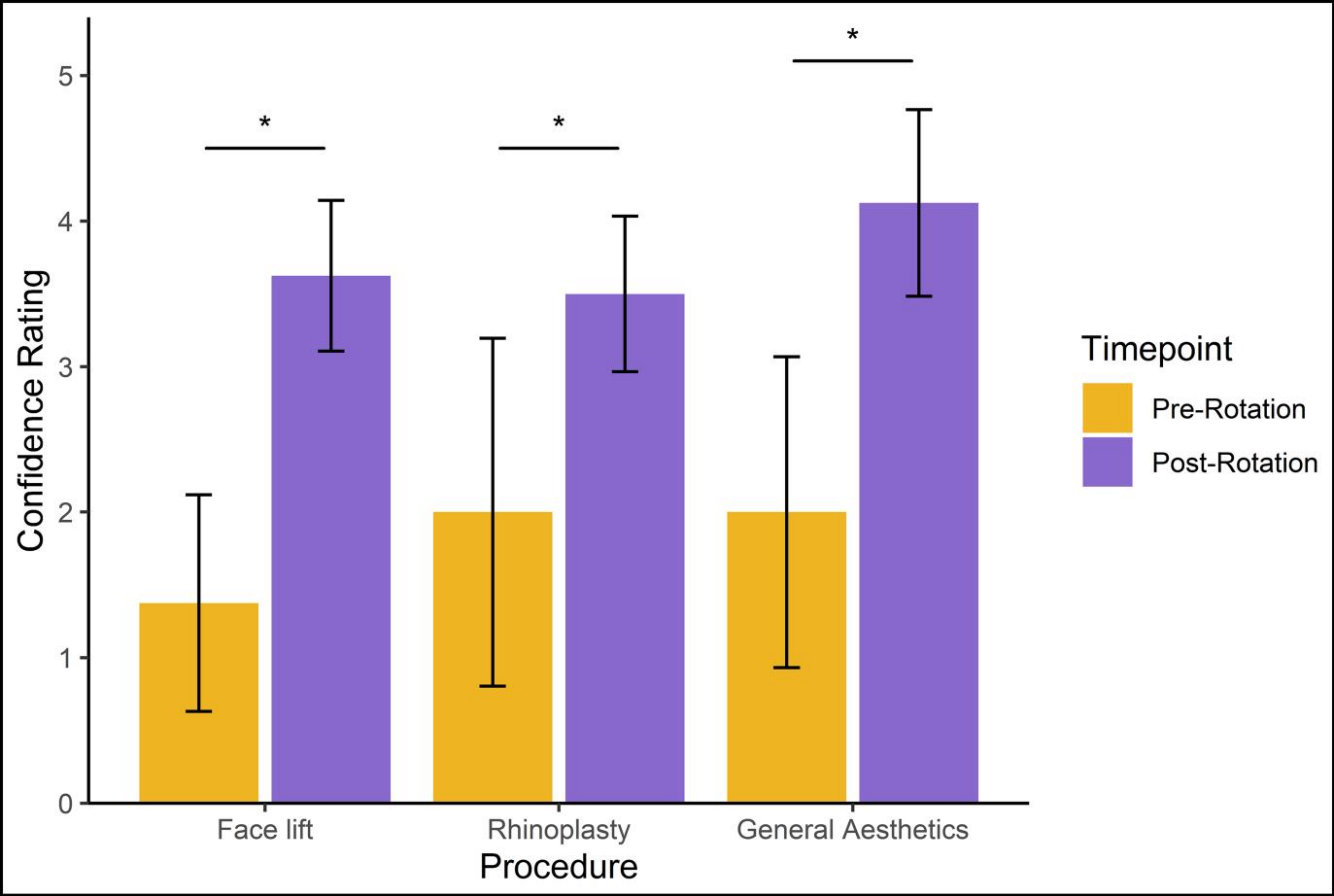
Quarterly variation of surgical case load with results of chi-square test for significance. **P < 0.01.

We compiled a total of 379 consultations corresponding to 237 unique patients who satisfied the inclusion criteria. Of these, we calculated a conversion rate of 38.0%, or 144 consultations resulting in an operation for 132 patients. Altogether, 420 distinct surgical procedures were performed, as we counted bilateral procedures as 2 separate procedures. When grouping bilateral procedures as 1 instead of 2, we totaled 293 procedures. Throughout the year, 12 attendings supervised these cases, with 2 attendings in particular dedicating substantial time to cover most of the cases. The vast majority of patients (111/132, 84.1%) underwent multiple procedures, though a smaller portion of patients (12/132, 9.1%) underwent a second aesthetic operation entirely (excluding revisions). The median time from the first consultation to surgery was 48 days. Throughout the 2021 calendar year, we observed significant quarterly variation in the number of procedures completed, with the highest number occurring in Q2 and the lowest occurring in Q3 (*P* < 0.01; Figure 2).

**Figure 3.**
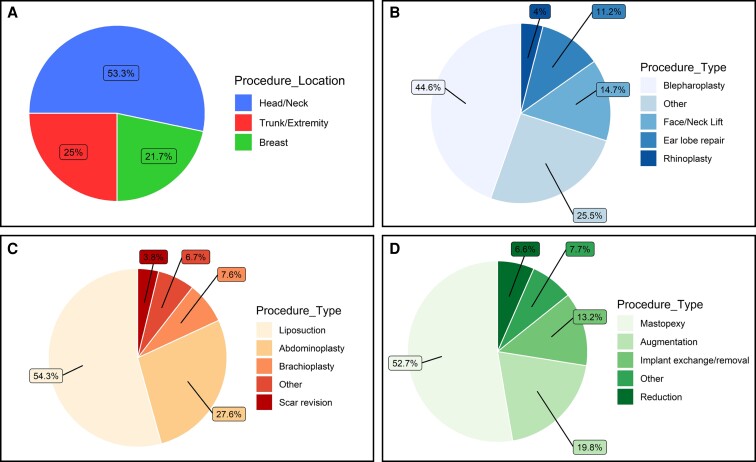
(A) Breakdown of 420 aesthetic procedures by body location. Breakdown of specific procedures performed on the (B) head and neck, (C) trunk and extremity, and (D) breast.

We then categorized procedures according to 3 body locations in accordance with ACGME graduating plastic surgery resident case requirements: head/neck, breast, and trunk/extremities. The majority of procedures involved the head and neck (53.3%), followed by the trunk and extremities (25.0%) and breast (21.7%) (Figure 3A). Blepharoplasty was the single most commonly performed procedure of the head and neck, accounting for nearly a quarter of all procedures in the clinic (Figure 3B; Table 2). For trunk/extremity procedures, liposuction and abdominoplasty were the most common (Figure 3C; Table 3). Lastly, mastopexy accounted for about half of all breast procedures, while a third of cases involved either breast augmentation or implant exchange/removal (Figure 3D; Table 4). Patients of the resident aesthetic clinic had a mean and median length of postoperative follow-up of 46.2 and 29 days, respectively (range 4-305 days).

**Table 2.** Head and Neck Procedures

Location	Procedure	No. (%)	No. (%)
Eye	Blepharoplasty	100 (44.6)	116 (51.8)
	Canthopexy	7 (3.1)	
	Brow lift	5 (2.2)	
	ORL release	2 (0.9)	
	Supraorbital burring	2 (0.9)	
Face	Face lift	17 (7.6)	46 (20.5)
	Mass/mole excision/shaving	10 (4.5)	
	Fat grafting	7 (3.1)	
	Vivace	4 (1.8)	
	Fat pad excision	4 (1.8)	
	Scar revision	3 (1.3)	
	Liposuction face	1 (0.4)	
Ear	Ear lobe repair	25 (11.2)	28 (12.5)
	Ear reduction	2 (0.9)	
	Ear augmentation with periauricular flap	1 (0.4)	
Neck	Neck lift	16 (7.1)	16 (7.1)
Nose	Rhinoplasty	9 (4)	11 (4.9)
	Alar base reduction	2 (0.9)	
Mouth	Chin implant	4 (1.8)	7 (3.1)
	Lip lift	1 (0.4)	
	Dynamic chin ptosis repair	1 (0.4)	
	Submandibular gland Botox	1 (0.4)	

Subcategorized into procedures of the eye, general face, ear, neck, nose, and mouth area. Total number of each procedure as well as percentage of all 224 head and neck procedures are shown. ORL, orbicularis retaining ligament.

**Table 3.** Trunk and Extremity Procedures

Location	Procedure	No. (%)	No. (%)
Abdomen	Abdominoplasty	29 (27.6)	70 (66.7)
	Liposuction abdomen	22 (21)	
	Liposuction flanks	17 (16.2)	
	Umbilical hernia repair	1 (1)	
	Umbilicoplasty	1 (1)	
Upper extremity (UE)	Brachioplasty	8 (7.6)	20 (19.0)
	Liposuction arms	8 (7.6)	
	Fat grafting UE	2 (1.9)	
	Liposuction axilla	2 (1.9)	
Lower extremity	Liposuction thighs	2 (1.9)	5 (4.8)
	Fat grafting buttock	2 (1.9)	
	BBL	1 (1)	
Back	Liposuction back	6 (5.7)	6 (5.7)
General	Scar revision	4 (3.8)	4 (3.8)

Subcategorized into procedures of the abdomen, upper extremity, lower extremity, back, and general trunk/extremity area. Total number of each procedure as well as percentage of all 105 trunk and extremity procedures are shown. BBL, Brazilian butt lift.

**Table 4.** Breast Procedures

Breast procedure	No. (%)
Mastopexy	48 (52.7)
Breast augmentation	18 (19.8)
Breast implant exchange/removal	12 (13.2)
Breast reduction	6 (6.6)
Fat grafting breast	4 (4.4)
NAC tattoo	2 (2.2)
Liposuction breast	1 (1.1)

Total number of each procedure as well as percentage of all 91 breast procedures are shown. NAC, nipple-areola complex.

### Procedures at the Aesthetic Surgery Clinic Yield Low Complication and Revision Rates

Of all 420 procedures, we report an overall complication rate of 5.5% (23/420 procedures). We report no major complications requiring immediate return to the operating room (Table 5). Most complications, including surgical site dehiscence and cellulitis, required nonoperative management. A total of 4 moderate complications occurred (1.0%), necessitating surgical intervention for management. Some of these complications, namely dog ears, lid ptosis, and dehiscence, contributed to our overall revision rate of 1.0% (4/420 procedures). Most revisions were to correct minor asymmetries and the presence of dog ears (Table 5). When compared with previously published data from other institutions,^8,9,11^ we found that our complication and revision rates were both lower. For transparency, when adjusting our calculations to reflect bilateral procedures being counted as a single procedure, we had a 7.8% complication rate (23/293 procedures) and 1.4% revision rate (4/293 procedures).

**Table 5.** Complication and Revision Rates

Complications			
Severity	Type	No. (%)	No. (%)
Minor	Dehiscence	10 (2.4)	19 (4.5)
	Ectropion	2 (0.5)	
	Cellulitis	2 (0.5)	
	Seroma	2 (0.5)	
	Neuropraxia upper extremity	1 (0.2)	
	Neuropraxia facial nerve branch	1 (0.2)	
	Partial umbilical skin necrosis	1 (0.2)	
Moderate	Seroma	1 (0.2)	4 (1.0)
	Dehiscence	1 (0.2)	
	Suture granuloma	1 (0.2)	
	Lid ptosis	1 (0.2)	
Major		0 (0)	0 (0.0)
Total complications		23 (5.5)	
Wake Forest 2000-2013^11^		6.1%	
WashU 2010-2015^9^		24.6%	
Revisions			
Category	Indication	No. (%)	No. (%)
Aesthetics	Asymmetry	1 (0.2)	2 (0.4)
	Dog ear	1 (0.2)	
Functional	Lid ptosis	1 (0.2)	1 (0.2)
Wound complication	Dehiscence	1 (0.2)	1 (0.2)
Total revisions		4 (1.0)	
Wake Forest 2000-2013^11^		12.8%	
UKentucky 1994-2004^8^		3.1%	
WashU 2010-2015^9^		2.9%	

Complications and revisions categorized by severity and revision reason, respectively. Total number of each complication and revision type shown, as well as contributing percentage to all complications and revisions. Rates compared to data from other resident aesthetic clinics.

### The Resident Aesthetic Clinic Provides Procedures at a Substantially Reduced Cost

An attractive aspect of the resident aesthetic clinic for patients is the reduced cost of procedures compared with private practice. We sought to compare our surgeon fees to the nationwide averages, as well as resident aesthetic clinics at other institutions. We compiled a list of 11 of our commonly provided procedures, categorized by body location. The surgeon fees for all 11 procedures at the NYU Resident Aesthetic Clinic were discounted 60% or more compared with the 2020 ASPS and 2021 Aesthetic Society national averages (Table 6). Moreover, nearly all of our surgeon fees were less than resident clinics at other institutions. Exceptions included blepharoplasty, liposuction, and brachioplasty.

**Table 6.** Surgeon Fees for Common Aesthetic Procedures

Location	Procedure	NYU average surgeon fee	2020 ASPS	Δ%	2021 Aesthetic Society	Δ%	UPenn 2009-2016 (Weissler et al 2017)	Δ%	Baylor 2018 (Wagner et al 2021)	Δ%
Head/neck	Blepharoplasty	$1122	$4120	−73%	$3963	−72%	$1800	−38%	$1063	6%
	Face lift	$1371	$8005	−83%	$9127	−85%	$2200	−38%	$1833	−25%
	Neck lift	$1345	$5774	−77%	$4167	−68%	$2200	−39%	$1500	−10%
	Rhinoplasty	$1031	$5483	−81%	$5443	−81%	$1800	−43%	n/a	
Trunk/extremity	Liposuction	$1107	$3637	−70%	$2736	−60%	$1700	−35%	$714	55%
	Abdominoplasty	$1153	$6154	−81%	$6764	−83%	$1850	−38%	$1835	−37%
	Brachioplasty	$1181	$4861	−76%	n/a		$1000	18%	$1300	−9%
Breast	Mastopexy	$1105	$5012	−78%	$4864	−77%	$1150	−4%	$2000	−45%
	Augmentation	$978	$4516	−78%	$4235	−77%	$2250	−57%	$1000	−2%
	Implant removal/exchange	$1142	$3049	−63%	$3161	−64%	n/a		n/a	
	Reduction	$1275	$5913	−78%	$5806	−78%	$1750	−27%	$2500	−49%

Average surgeon fees calculated for 11 common aesthetic procedures. Fees compared to national averages and data from other resident aesthetic clinics. Percent changes depict relative discount of NYU surgeon fees compared to literature data.

### Aesthetic Training Improves Resident Confidence in Performing Aesthetic Procedures

**Figure 4.**
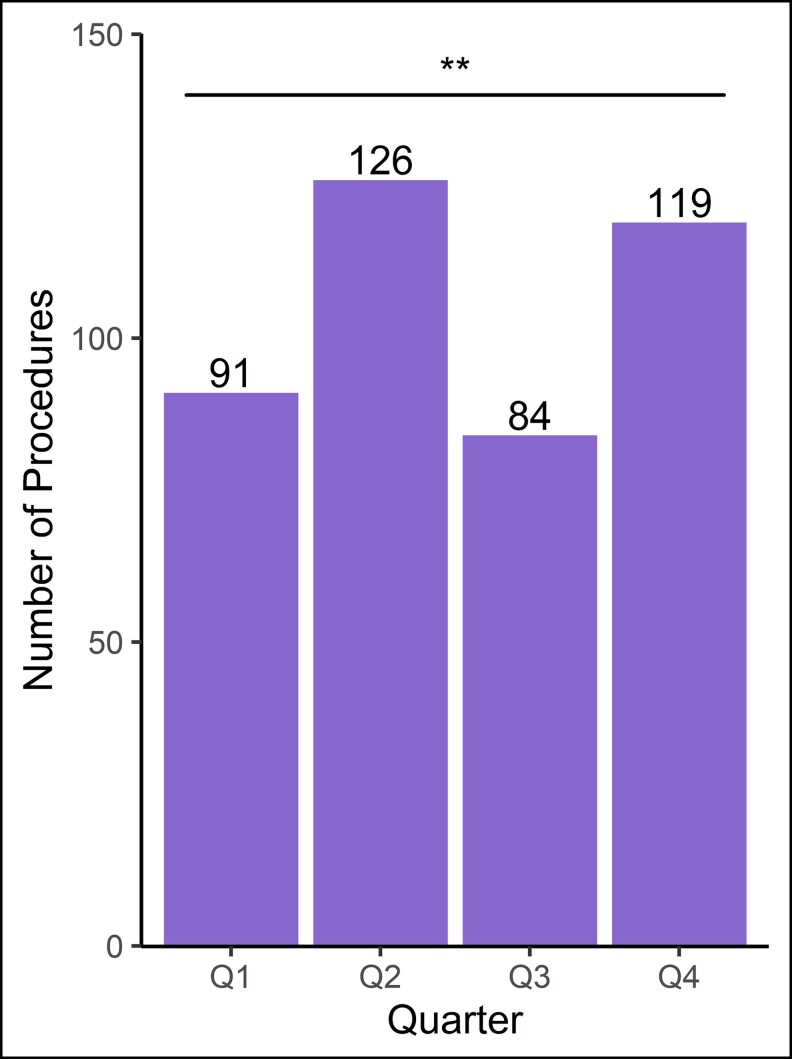
Residents’ confidence ratings for face lift, rhinoplasty, and aesthetic surgery pre- and postrotation with results of the Mann–Whitney test for significance. *P < 0.05.

We first calculated the average number of procedures performed by each resident year. Since residents’ postgraduate year changes in the middle of the calendar year, we grouped residents by graduating class for the most consistency. On average, residents from any postgraduate year were involved in 78 procedures. Residents in the graduating class of 2022 (PGY-5/6 during 2021) averaged 109 procedures (Supplemental Table 2). To understand the subjective impact of the aesthetic clinic on trainees, participating residents rated their confidence in performing aesthetic procedures before and after their clinic rotation. We found that PGY-5 and PGY-6 residents were significantly more confident performing face lifts, rhinoplasties, and aesthetic surgery in general after rotating in the clinic (*P* < 0.05; Figure 4). In addition, postrotation confidence ratings had much less variability compared with prerotation scores.

## DISCUSSION

In this study, we evaluated surgical outcomes, costs, and resident perspectives of the Resident Aesthetic Surgery Clinic at NYU Langone Health in the 2021 calendar year. The vast majority of patients presenting to the clinic were female in relatively good health, exhibiting low rates of diabetes, active tobacco use, and obesity (Table 1). The aesthetic clinic demonstrated marked productivity and efficiency with 420 total procedures (Figure 2), including 224 of the head and neck (Table 2), 105 of the trunk and extremities (Table 3), and 91 of the breasts (Table 4). Even in the setting of such a high volume, residents under attending supervision demonstrated excellent technical skill with an overall complication rate of 5.5% and an overall revision rate of 1.0%, lower than rates reported by several other institutions^8,9,11^ (Table 5). Our resident aesthetic clinic continues to provide affordable care for patients, with surgeon fees drastically lower than national averages^1,13^ and lower than most other institutions^10,12^ (Table 6). Altogether, we have shown that our resident aesthetic clinic is a robust training model for improving aesthetic surgery exposure, competence, and confidence of plastic surgery residents (Figure 4).

For many years, there has been a discrepancy between the rising public demand for aesthetic procedures and the training of plastic surgery residents. As a result, there is a need to reevaluate residency training models to ensure that graduating residents are prepared to incorporate aesthetic surgery in their future practice. There has been a surge of studies analyzing the surgical outcomes of resident aesthetic clinics and surveying the impressions of participating residents. We report the largest annual study to date with 420 total procedures in the 2021 calendar year. Other institutions have typically reported their findings over several years, averaging to 44-81 procedures per year.^8,12,14^ This discrepancy in output may be partially attributable to our definition of a procedure; however, it is difficult to compare to previously reported studies as they do not transparently define what was counted as a procedure. Under our definition, bilateral surgeries were counted as 2 separate procedures. In addition, blepharoplasties were also separated by upper and lower lids. This definition may lend itself to overestimating the case load of the clinic, while underestimating its complication and revision rates. Nevertheless, grouping all bilateral surgeries as 1 procedure still totaled to 293 procedures across the year, substantially higher than previously published data. This clinical volume is a testament to the resident aesthetic clinic model at our institution which depends on the collaborative efforts of residents, attendings, administration, and clinical staff.

Plastic surgery residents have consistently felt less confident in facial aesthetics, specifically face lifts and rhinoplasties.^15^ This can be a difficult obstacle to overcome because the most common procedures at resident aesthetic clinics typically involve the body or breast rather than the face.^11,12^ By contrast, at our clinic, we performed head and neck aesthetic procedures at a higher frequency than breast or body cases. In total, our clinic performed 17 face lifts and 9 rhinoplasties during 2021. The increase in our residents’ confidence is also likely due to their sheer individual case volume and autonomy in the clinic. PGY-5/6 residents were involved in 109 cases on average, similar to the University of Kentucky's reported 105 cases per chief resident.^8^ Our case distribution demonstrates the value that this approach to resident aesthetic training provides by augmenting their exposure to facial aesthetics under the expertise of attending surgeons.

Rates of complications and revisions can be used as a proxy to judge the safety and efficacy of aesthetic procedures. We reported a low complication rate of 5.5%, with no major complications among the 420 procedures completed. Of the 1.5 years of data collected to date in this ongoing study, there has only been 1 major complication requiring return to the OR. Our anecdotal experience throughout the years corroborates this as well. Major complications have been quite infrequent, and the year-long complication rate presented in this study is not anomalous to previous years. Using statistics from CosmetAssure (Birmingham, AL), an insurance program that covers selected aesthetic procedures performed by members of the American Society of Plastic Surgeons (ASPS), we can compare our rates to national benchmarks. It is important to note that CosmetAssure only reports complications requiring hospitalization, emergency room care, or surgical center intervention within 30 days postoperatively,^16^ thus minor complications (under our definition) are not captured. This repository reports a complication rate of 1.64%,^16^ which is comparable to our combined moderate/major complication rate of 1%. Our overall complication rate was the lowest of all other institutions that we evaluated, demonstrating the safety of procedures performed by residents under attending supervision. Our revision rate of 1.0% is also the lowest reported to date. Most of the revisions were due to immediate aesthetic reasons rather than complications, further emphasizing the safety of our clinic for patients. This feat is accredited to the resident training model as a whole, including direct care from residents in conjunction with input from expert attendings, perioperative care by nursing and anesthesia, and logistical support from administrators.

Equally important as evaluating the surgical success of resident aesthetic clinics is evaluating their affordability for patients. Compared with nationwide surgeon fee averages,^1,13^ we have shown that the NYU Resident Aesthetic Surgery Clinic offers procedures at substantially reduced prices. Compared with other institutions,^10,12^ our surgeon fees were nearly all less expensive. There were some exceptions though, namely blepharoplasties, liposuction, and brachioplasties which had slightly higher surgeon fees compared with another institution. Despite these slightly higher costs, it is important to keep in mind that the surgeon fees of other institutions were not adjusted for inflation or an average annual increase in surgeon fees throughout the years. Surgeon fees typically increase over time, even up to 49% year to year.^1^ Altogether, our comparison to other institutions is conservative and likely underestimates the discount of our surgeon fees.

An interesting trend we discovered was the significant seasonal variability of procedures in the aesthetic clinic, with our highest caseload during Q2 and lowest during Q3. There have been limited analyses that have described the seasonal demands for aesthetic procedures to date. One study, however, suggested a relatively increased demand for aesthetic procedures during Ramadan in Saudi Arabia, citing reasons such as upcoming summer weddings and summer vacations.^17^ Our results are not well explained and likely do not reflect long-term seasonal trends, especially during the COVID-19 pandemic. Nonetheless, investigating seasonal trends in aesthetic surgery case load may be valuable for determining patient motivations for seeking aesthetic surgery. Further studies incorporating several years of data are required to fully understand these seasonal variations.

This study is not without limitations. Despite our surgeon fees being quite discounted, this does not reflect the total cost of the procedure to the patient. Anesthesia and operating room fees often account for the majority of the procedure cost (typically ranging from $4000 to 7000 at our institution), which were not discounted or evaluated here. Unfortunately, data on these fees are not readily available in the literature or national databanks, and can vary between institutions and geographic regions. Similarly, it is vital to publish information regarding the cost to sustain a resident aesthetic clinic, as this is beneficial for other institutions looking to inaugurate their own. Operational costs, however, are entirely dependent on market conditions, the structure of an existing plastic surgery unit, and geography. Such conditions are not able to be quantified to apply broadly and were not the focus of this study. We do outline the minimum components though, namely a dedicated full-time coordinator, a functioning outpatient operating room suite with staff and anesthesia support, as well as faculty who allot excusive time/effort to supervise the clinic. Other limitations of our study pertain to standardization. Standard definitions for major and minor complications do not currently exist; instead, subjective measures are used to classify the severity of surgical complications. Furthermore, complications may be restricted to different time intervals (eg, 30, 45 days, etc.) across studies, while we considered complications regardless of postoperative day. Similarly, our follow-up time was limited by the relatively short duration of our study, which may contribute to our low rate of revisions and long-term complications. Indeed, our revisions were mostly related to correcting complications or other imperfections that are salient within weeks/months after the index procedure. Revisions due to cosmetic dissatisfaction can occur several months or even years later, as 1 study found a median time to septorhinoplasty revision of 1.2 years.^18^ These revisions may not be exclusively due to cosmetic reasons though, as most of the index procedures in this study were due to functional reasons. Another bias that we cannot exclude, nor adequately control for, is the possibility of patients transferring care to another institution/provider outside of NYU. Future studies aim to mitigate our follow-up time bias by including several years of aesthetic clinic data.

Future studies should incorporate methods to combat the limitations we have described. It would be advantageous to publish data on anesthesia and operating room fees in order to better understand the variability between regions and institutions. More detailed and clearer definitions of what constitutes a procedure and complication should be stated as well. It would also be beneficial to administer a more detailed standardized survey to residents before and after their aesthetic clinic rotation, and correlate to their objective metrics of performance. These changes are necessary to more accurately evaluate the educational benefits and productivity of resident aesthetic surgery clinics.

## CONCLUSIONS

As the demand for aesthetic surgery rises, it is necessary to ensure adequate training for plastic surgery residents by evaluating current training modalities. This study demonstrates the training model and experience of our Resident Aesthetic Surgery Clinic at NYU Langone Health. We report a very high surgical output, specifically for head and neck procedures, while maintaining a low complication rate, revision rate, and cost to patients. Our results provide evidence that resident aesthetic surgery clinics are a safe and efficacious training modality for plastic surgery residents to refine their skills and improve confidence in aesthetic surgery through the collaborative efforts and support from attendings, administration, and clinical staff.

## Supplementary Material

ojac084_Supplementary_Data
