# Correction to: Productivity and Efficiency of a Department Resident Aesthetic Plastic Surgery Clinic

**DOI:** 10.1093/asjof/ojad011

**Published:** 2023-02-06

This is a correction to: Hani Y Nasr, MD, Carter J Boyd, MD, MBA, Zachary M Borab, MD, Neil M Vranis, MD, Michael F Cassidy, BA, Alexis K Gursky, BS, Rebecca Gober, BA, Barry M Zide, MD, DMD, Daniel J Ceradini, MD, Productivity and Efficiency of a Department Resident Aesthetic Plastic Surgery Clinic, *Aesthetic Surgery Journal Open Forum*, Volume 4, 2022, ojac084, https://doi.org/10.1093/asjof/ojac084

In the originally published version of this manuscript the images used for figures two and four were inadvertently swapped. There is no change to the associated captions.

This error, for which the publisher apologizes, has been corrected.

