# Supplementary material for: Productivity and Efficiency of a Department Resident Aesthetic Plastic Surgery Clinic
Source: Aesthet Surg J Open Forum. 2022 Dec 6;4:ojac084. doi: 10.1093/asjof/ojac084 (PMC9750105; doi:10.1093/asjof/ojac084)
Supplement: ojac084_Supplementary_Data [file ojac084_supplementary_data.zip › 22-0104_Supplemental Table 1.docx]

**Supplemental Table 1:** Resident Confidence Questionnaire

| Questions | Responses |
| --- | --- |
| How confident are you performing aesthetic surgery? | 1 = not at all confident 2 = slightly confident 3 = somewhat confident 4 = fairly confident 5 = completely confident |
| How confident are you performing face lift surgery? |  |
| How confident are you performing rhinoplasty surgery? |  |

Three questions posed to residents in a survey completed before and after their aesthetic clinic rotation.
