# Supplementary material for: Productivity and Efficiency of a Department Resident Aesthetic Plastic Surgery Clinic
Source: Aesthet Surg J Open Forum. 2022 Dec 6;4:ojac084. doi: 10.1093/asjof/ojac084 (PMC9750105; doi:10.1093/asjof/ojac084)
Supplement: ojac084_Supplementary_Data [file ojac084_supplementary_data.zip › 22-0104_Supplemental Table 2.docx]

**Supplemental Table 2:** Procedures Performed per Resident

| Graduating class | Avg. head/neck (range) | Avg. trunk/extremities (range) | Avg. breast (range) | Avg. total Ppocedures (range) |
| --- | --- | --- | --- | --- |
| 2023 (PGY-4/5) | 26 (12-25) | 18 (7-26) | 14 (0-22) | 58 (9-67) |
| 2022 (PGY-5/6) | 59 (49-68) | 28 (10-43) | 22 (11-40) | 109 (87-142) |
| 2021 (PGY-6/graduated) | 37 (16-58) | 15 (3-26) | 13 (0-22) | 64 (34-93) |

Average number of procedures performed by individual residents, split by graduating class and procedure category. PGY, postgraduate year.
